# Tincture of Benzoin for Chapped Nipple

**Published:** 1861-01

**Authors:** 


*Tincture of Benzoin for Chapped Nipple.*—Some years ago Mr. Bourdel extolled the virtues of tincture of benzoin for the cure of excoriated or chapped nipple. The remedy was applied with a camel's hair pencil, immediately after suckling the child, or oftener, if necessary, and in such a manner as to cover the sore parts with a uniform layer of the liquid.

I have also, says Mr. Moulas, of Douai, since 1854 had recourse to the method recommended by Mr. Bourdel, and have found the tincture of benzoin constantly successful in the cases alluded to. This remedy is particularly valuable, as in the first place the child takes the breast without repugnance; in the second, the tincture leaves, on evaporating, a layer of benzoin on the surface of the nipple, thus affording to the sores protection from contact with the air or with the dress; and lastly the nurse can give the breast without previously washing the nipple, an operation which induces much pain.—(*Championniere's Journal.*)

